# Comparison of treatment effect sizes from pivotal and postapproval trials of novel therapeutics approved by the FDA based on surrogate markers of disease: a meta-epidemiological study

**DOI:** 10.1186/s12916-018-1023-9

**Published:** 2018-03-21

**Authors:** Joshua D. Wallach, Oriana Ciani, Alison M. Pease, Gregg S. Gonsalves, Harlan M. Krumholz, Rod S. Taylor, Joseph S. Ross

**Affiliations:** 1 Collaboration for Research Integrity and Transparency (CRIT), Yale Law School, 157 Church Street, 17th Floor, Suite 1, New Haven, CT 06510 USA; 2 Center for Outcomes Research and Evaluation (CORE), Yale–New Haven Hospital, 1 Church Street, Suite 200, New Haven, CT 06510 USA; 3 Evidence Synthesis and Modelling for Health Improvement, Institute of Health Research, University of Exeter Medical School, South Cloisters, St. Luke’s Campus, Heavitree Road, Exeter, EX1 2LU UK; 4 Center for Research on Health and Social Care Management, SDA Bocconi, via G. Roentgen, 1 - 20136, Milan, Italy; 5 Department of Surgery, Beth Israel Deaconess Medical Center, Lowry Medical Office Building, 110 Francis Street, Suite 9B, Boston, MA 02215 USA; 6 Department of Epidemiology of Microbial Diseases, Yale School of Public Health, 60 College Street, P.O. Box 208034, New Haven, CT 06520-8034 USA; 7 Section of Cardiovascular Medicine, Department of Internal Medicine, Yale School of Medicine, New Haven, CT 06520-8092 USA; 8 Department of Health Policy and Management, Yale School of Public Health, 60 College Street, New Haven, CT 06510 USA; 9 Section of General Medicine, Department of Internal Medicine, Yale School of Medicine, P.O. Box 208093, New Haven, CT 06520-8093 USA

**Keywords:** Surrogate markers, Outcomes, Lifecycle evaluation, Regulation, U.S. Food and Drug Administration (FDA)

## Abstract

**Background:**

The U.S. Food and Drug Administration (FDA) often approves new drugs based on trials that use surrogate markers for endpoints, which involve certain trade-offs and may risk making erroneous inferences about the medical product’s actual clinical effect. This study aims to compare the treatment effects among pivotal trials supporting FDA approval of novel therapeutics based on surrogate markers of disease with those observed among postapproval trials for the same indication.

**Methods:**

We searched Drugs@FDA and PubMed to identify published randomized superiority design pivotal trials for all novel drugs initially approved by the FDA between 2005 and 2012 based on surrogate markers as primary endpoints and published postapproval trials using the same surrogate markers or patient-relevant outcomes as endpoints. Summary ratio of odds ratios (RORs) and difference between standardized mean differences (dSMDs) were used to quantify the average difference in treatment effects between pivotal and matched postapproval trials.

**Results:**

Between 2005 and 2012, the FDA approved 88 novel drugs for 90 indications based on one or multiple pivotal trials using surrogate markers of disease. Of these, 27 novel drugs for 27 indications were approved based on pivotal trials using surrogate markers as primary endpoints that could be matched to at least one postapproval trial, for a total of 43 matches. For nine (75.0%) of the 12 matches using the same non-continuous surrogate markers as trial endpoints, pivotal trials had larger treatment effects than postapproval trials. On average, treatment effects were 50% higher (more beneficial) in the pivotal than the postapproval trials (ROR 1.5; 95% confidence interval CI 1.01–2.23). For 17 (54.8%) of the 31 matches using the same continuous surrogate markers as trial endpoints, pivotal trials had larger treatment effects than the postapproval trials. On average, there was no difference in treatment effects between pivotal and postapproval trials (dSMDs 0.01; 95% CI -0.15–0.16).

**Conclusions:**

Many postapproval drug trials are not directly comparable to previously published pivotal trials, particularly with respect to endpoint selection. Although treatment effects from pivotal trials supporting FDA approval of novel therapeutics based on non-continuous surrogate markers of disease are often larger than those observed among postapproval trials using surrogate markers as trial endpoints, there is no evidence of difference between pivotal and postapproval trials using continuous surrogate markers.

**Electronic supplementary material:**

The online version of this article (10.1186/s12916-018-1023-9) contains supplementary material, which is available to authorized users.

## Background

The United States Food and Drug Administration (FDA) review of new drug applications is guided by the *Federal Food, Drug, and Cosmetic Act*, which suggests that drug manufacturers submit “adequate and well-controlled” trials providing evidence of drug safety and efficacy for marketing approval (i.e., pivotal efficacy trials) [1]. Between 2002 and 2012, 49% of pivotal trials supporting approved new drug indications used surrogate markers of disease as primary endpoints (i.e., biomarkers or intermediate endpoints) that were deemed “reasonably likely” to predict patient-relevant outcomes (e.g., mortality and morbidity) [1, 2]. Furthermore, the majority of pivotal trials enrolled fewer than 1000 patients with a follow-up of 6 months or less [2].

The use of surrogate markers of disease as primary endpoints can shorten the duration, size, and thus the total financial cost of a pivotal trial [3–5]. However, trials that use surrogate markers for endpoints also involve certain trade-offs and may risk making erroneous inferences and have diminished certainty about the medical product’s actual clinical effect. Recent studies have shown that commonly used surrogate markers can be poor proxies for patient-relevant outcomes [6–8]. This dilemma is reflected in recommendations for cautious use of surrogate markers in drug assessment by many international health technology appraisal agencies [9]. In addition, shorter trials or trials that have been terminated early can lead to biased treatment effects due to large random fluctuations of the estimated treatment effect that can occur over shorter observation periods [10, 11]. Similarly, smaller trials may be more likely to result in findings that appear more favorable compared with true treatment effects [12–15]. Other dimensions of lower methodological quality, including lack of double-blinding, have also been associated with larger treatment effects [16].

Perceptions of the potential treatment benefits of novel drugs are often based on the results from pivotal trials used during the FDA approval process. Evidence suggests that the quantity and quality of postapproval trial evidence varies substantially for drugs approved by the FDA based on pivotal trials that use surrogate markers of disease as primary endpoints [17]. Considering that there is growing pressure for shorter regulatory evaluations and wider adoption of surrogate markers as part of clinical trial design, it is necessary to determine whether the evidence used to support the approval of novel therapeutics by the FDA is reliable and potentially consistent in postapproval trials evaluating the same drugs in diverse populations.

The objective of this study was to compare the treatment effects among pivotal trials supporting FDA approval of novel therapeutics between 2005 and 2012 based on surrogate markers of disease and the treatment effects observed among postapproval trials evaluating the same surrogate markers of disease. We also aimed to determine whether the treatment effects among pivotal trials with surrogate markers as primary endpoints were consistent with the treatment effects from postapproval trials using patient-relevant outcomes. Results from this work will inform regulatory decision-making and efforts to promote the adoption of surrogate markers as part of clinical trial design.

## Methods

The protocol for this study was pre-specified prior to performing any data analysis (see Additional file 1).

### Identification of approved therapeutics

We used previously collected data on novel therapeutics first approved by the FDA between 1 January 2005 and 31 December 2012 [2]. We did not consider additional novel therapeutics approved after 31 December 2012 because insufficient time has passed since approval to allow for completion and publication of postapproval trials. The Drugs@FDA database was used to categorize each novel therapeutic agent by year of approval and as a pharmacologic entity (small molecule) or biologic. FDA approval letters, which are hyperlinked in the Drugs@FDA database, were then used to determine the indications for which all novel therapeutic agents were initially approved for use, whether agents were orphan drugs, whether agents were approved through the accelerated approval pathway, and whether applications were designated by the FDA for priority or standard review. The World Health Organization’s Anatomic Therapeutic Classification system was used to categorize each indication into therapeutic areas (cancer, cardiovascular disease and diabetes mellitus, infectious disease, and other) [18].

### Identification of pivotal trials and primary trial endpoints

Pivotal trials were those labeled in FDA medical reviews as “pivotal.” If FDA did not clearly specify the pivotal trials, then trials that were described as essential to approval, were prioritized in the review, or were new efficacy based trials provided as part of a resubmitted application for approval were classified as pivotal [2, 17]. Primary trial endpoints were classified as patient-relevant outcomes (e.g., mortality and morbidity), which represent patient survival or function, or surrogate markers of disease (e.g., changes in blood pressure), which represent biomarkers expected to predict clinical benefit, based on an established framework and an Institute of Medicine report [2, 19, 20]. Only therapeutics that were approved exclusively based on a surrogate marker were included in this evaluation. Study descriptions, additional definitions, and inclusion and exclusion criteria have been described in prior research [2].

### Identification of pivotal trial publications

Publications of pivotal trials for novel therapeutic agents approved between 2005 and 2011 have been described in previously published research [21]. Briefly, the Scopus database (Elsevier Inc.) was searched for the period from April through October 2012 using the terms “[generic drug name]” AND “clinical trial.” When necessary, the manufacturer-designated trial identification numbers of six or more characters were entered into the advanced search feature of ClinicalTrials.gov. As described in prior research, four criteria were used to identify matching publications: study design, indication, intervention, and intention-to-treat enrollment [21]. One author (JDW) replicated this search process to locate publications for the novel therapeutic agents approved in 2012.

### Identification of postapproval trials

The international non-proprietary name of each drug was searched for in Medline by two investigators to locate all postapproval prospective studies in humans that used an active or placebo control as a comparator arm and examined efficacy for the same therapeutic indication for which the drug was originally approved by the FDA, as described in previous work [17]. The primary trial endpoints of eligible postapproval trials were then classified as patient-relevant outcomes, clinical scales, or surrogate markers using the previously described established framework and a recent Institute of Medicine report [2, 19, 20]. Medline was utilized because it is the largest database of biomedical journal articles that can be searched freely using the PubMed system. Furthermore, the vast majority of clinicians and policymakers rely on the PubMed system to learn about clinical trial findings. Study descriptions, additional definitions, and inclusions and exclusion criteria have been described in prior research [17].

### Study sample

One author (JDW) applied the inclusion and exclusion criteria to all identified pivotal and postapproval trials. We excluded pivotal and postapproval trials that (1) were not published, (2) were not interventional randomized trials, (3) had an equivalence or non-inferiority design, (4) had only one arm (i.e., no comparator groups) or evaluated only different dosages of the same drug, (5) were crossover trials, or (6) had no analyzable data. We excluded postapproval trials that only had treatment arms where the novel therapeutic of interest was combined with other active interventions not considered in any of the corresponding pivotal trials. Although individual pivotal trial results are available in the FDA medical reviews on the Drugs@FDA database, our study focused on the pivotal trial data published in peer-reviewed biomedical journals. This allowed for the construction of matched pairs of published pivotal and postapproval trials. Doctors and policymakers often depend on the results from published clinical trials. As such, published pivotal trials reflect the data available during the FDA approval process and are a source that can be used to inform and influence real-world clinical practice. Further justifying this decision, nearly 90% of pivotal trials are published in the peer-reviewed biomedical literature [21]. Potential matches and uncertainties were discussed with an additional investigator (JSR).

### Matching pivotal trials with postapproval trials

To create a sample of comparable published pivotal and postapproval trials, one investigator (JDW), in discussion with two others (OC and JSR), developed and undertook a pre-specified matching process to pair each pivotal trial for each drug and approved indication with one postapproval randomized controlled trial. Prior to data collection, we decided to match pivotal and postapproval trials individually. Although multiple pivotal and postapproval trials could have been pooled and then compared, we suspected that matching individual trials would help simplify our analysis while also maximizing the likelihood of identifying high-similarity matches between pivotal and postapproval trials. We established a hierarchical matching process based on a pervious meta-epidemiological study comparing groups of trials using surrogate and patient-relevant outcomes [6]. The vast majority of potential matches were discussed with and reviewed by an additional investigator (JSR).

#### Postapproval trials using surrogate markers

When postapproval trials used surrogate markers for a trial endpoint, a successful match of a pivotal and postapproval trial was based on the following hierarchical matching process: use of (1) the same novel therapeutic for the same indication, (2) the same surrogate marker that was the primary outcome in the pivotal trial(s) used to form the exclusive basis of approval by the FDA, (3) the same intervention dosage, and (4) the same comparator. To ensure there was an adequate number of potential matches, we did not require similar population demographic and clinical characteristics. To maximize the number of potential matches, pivotal and postapproval trials using surrogate markers were required only to evaluate the same drug for the same indication with the same endpoint based on the same surrogate marker (criteria 1 and 2). For criterion 2, we allowed some flexibility in terms of timing (e.g., a pivotal trial using a primary endpoint of sustained virologic response at week 24 could be matched with a postapproval trial using sustained virologic response at week 12) and how the outcomes were measured (e.g., the time of day a measurement was taken). For dosage, we looked for treatment arms in the pivotal and postapproval trials with the exact same therapeutic dosage (e.g., a pivotal trial evaluating 750 mg of telaprevir twice a day could be matched with a postapproval trial evaluating 1500 mg once a day), but did not require the timing of the treatment (e.g., multiple injections provided 7–9 h apart) or the length of treatment (e.g., 12 weeks versus 24 weeks) to be exactly the same. We allowed some flexibility in terms of background therapies when matching pivotal and postapproval trials (e.g., a pivotal trial evaluating liraglutide in combination with metformin and thiazolidinedione could be matched with a postapproval trial evaluating liraglutide in combination with metformin only). When possible, we matched pivotal and postapproval trials that used the same comparator arm. When pivotal trials had only a placebo comparator and postapproval trials had only active comparators, we selected the comparator arm in the postapproval trial with the lowest dosage. When the pivotal and postapproval trials were multi-armed, we selected the intervention and comparator arms that were the most similar. When pivotal trials had multiple postapproval trial matches, we selected the trial with the longest follow-up time and the largest number of intention-to-treat patients in the intervention and comparator arms, as these trials would be expected to bring more reliable results.

#### Postapproval trials using patient-relevant outcomes (exploratory analyses)

When postapproval trials used patient-relevant outcomes for the primary or secondary trial endpoint, a successful match of a pivotal and postapproval trial required that they each evaluated the same drug for the same indication. For potential matches, we further identified whether the matched trials evaluated the same intervention dosage and the same comparator (i.e., placebo, usual care, or active comparator).

### Data extraction

For pivotal and postapproval trials, we recorded: total sample size (intention to treat: all subjects initially randomized, or modified intention to treat: all subjects randomized who received at least one treatment), trial duration (in weeks), blinding (double or triple blind, single blind or open label), comparator arms included in the trial (placebo only, active only, or both), center status (multicenter or single center), funding (for profit, not for profit, mixed, or none), and certain demographic characteristics (percentage female, percentage non-Caucasian, and mean or median age of study subjects). We also extracted the number of patients and events in the selected treatment and control arms, the absolute or relative effect sizes, confidence intervals (CIs), standard deviations, standard errors, or any other available data used to calculate the endpoints based on surrogate markers or patient-relevant outcomes. When necessary, an online digitizer (Web-PlotDigitizer; https://automeris.io/WebPlotDigitizer/) was used to extract approximate values from figures. Lastly, we recorded whether the matched trial pairs using surrogate markers fulfilled two, three, or four of the matching criteria described above.

### Data analyses

We used descriptive statistics to characterize the eligible novel drugs approved by the FDA and to summarize the design features of the pivotal trials and matched postapproval trials. We used Wilcoxon’s signed rank and McNemar’s exact tests to examine differences between matched pairs. Descriptive analyses were performed using R (version 3.4.0; The R Project for Statistical Computing) and meta-analyses were performed using the metafor package in R [22]. All statistical tests were two tailed and used a type 1 error rate of 0.05.

#### Calculation of treatment effects

Standardized mean differences (Cohen’s *d*) were calculated for all pivotal trials reporting continuous endpoints. For all pivotal and postapproval trials with endpoints reporting counts, proportions, or relative effect estimates (hereafter, non-continuous endpoints), we calculated odds ratios or approximated them from other relative risk measures provided. Similar to previous meta-epidemiological evaluations, we assumed that reported relative risks or hazard ratios were approximations to the odds ratio [6, 23]. The direction of effect was standardized so that an odds ratio above 1.0 and a positive standardized mean difference (greater than 0.0) indicated a beneficial effect of intervention compared to comparator arms.

#### Primary analyses

When pivotal trials were matched only to a postapproval trial using surrogate markers of disease for one of the trial endpoints, we first separately combined the standardized mean differences and odds ratios across pivotal and postapproval trials using the DerSimonian and Laird procedure for random effects. For each matched pair with a continuous endpoint based on a surrogate marker, we then estimated paired differences between standardized mean differences. For each matched pair with non-continuous surrogate markers of disease as trial endpoints, we converted odds ratios to natural log odds ratios and then calculated the ratio of odds ratios. A positive difference between standardized mean differences (greater than 0.0) and a ratio of odds ratios greater than 1.0 implied greater (more beneficial) treatment effects in the pivotal trials using a surrogate marker than in the postapproval trials using a surrogate marker. Considering that individual pivotal and postapproval trials were matched based on two to four criteria, we calculated the variance of each individual difference between standardized difference and ratio of odds ratio using two methods: (1) assuming that the pivotal and postapproval trials in the matched pairs were independent and (2) assuming between-study correlations of 0.5. If different results were observed using the two methods, we planned to repeat the calculations using correlation coefficients of 0.2, 0.3, and 0.4. If similar results were observed using the different variance approximations, we reported the more conservative estimates (assuming that all matched pivotal and postapproval trials were independent). Differences between standardized mean differences and ratios of odds ratios were separately combined using the DerSimonian and Laird procedure for random effects. We performed our analyses under the random-effects meta-analysis model assumptions. In particular, we assumed that the true treatment effect might be different between individual trials (e.g., treatment effects could be higher among trials with older or less healthy patients) [24]. All analyses were repeated for pivotal and postapproval trial matches that fulfilled at least three or all four of the matching criteria.

#### Secondary analyses

Standardized mean differences and associated variances for all pivotal and postapproval trials reporting continuous endpoints were transformed to natural log odds ratios [25]. The ratio of odds ratios from all matched pairs fulfilling two, three, or all four matching criteria were then combined using the DerSimonian and Laird procedure for random effects.

#### Exploratory analyses

When pivotal trials were matched to postapproval trial using patient-relevant outcomes for one of the trial endpoints, the standardized mean differences from the pivotal trials were transformed to natural log odds ratios [25]. We then calculated the ratio of odds ratios. The paired ratios of odds ratios were then combined using the DerSimonian and Laird procedure for random effects. Ratios of odds ratios greater than 1.0 implied greater (more beneficial) treatment effects in the pivotal trials than in the postapproval trials. All variances were calculated as described above.

### Patient involvement

No patients were involved in setting the research question or the outcome measures, nor were patients involved in any other aspect of study design or implementation.

## Results

### Sample characteristics

**Fig 1:**
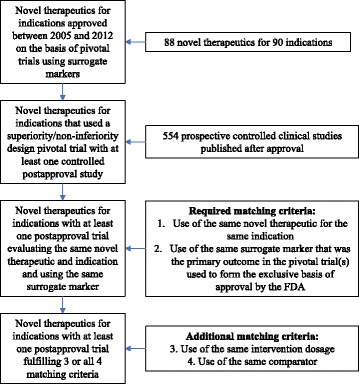
Sample construction for pivotal trials using surrogate markers with matched postapproval trials using surrogate markers

Between 2005 and 2012, the FDA approved 88 novel drugs for 90 indications based on one or multiple pivotal trials using surrogate markers of disease. However, not all indications had at least one published controlled postapproval study using an active or placebo control that examined efficacy for the same indication for which the drug was first approved. In total, we identified 34 eligible indications with a total of 93 published pivotal trials that used a superiority or a superiority/non-inferiority design and had at least one controlled postapproval study (Fig. 1).

#### Postapproval trials using surrogate markers

We located 27 novel drugs for 27 indications based on pivotal trials using surrogate markers as primary endpoints that could be matched to postapproval trials evaluating the same drug and indication and using the same surrogate markers as endpoints, for a total of 43 matches. The majority of the 43 matched trial pairs (one pivotal trial matched to one postapproval trial) fulfilled all four of the hierarchical matching criteria (33 of 43). Only four (9.3%) had matched trials with different dosages and comparator arms. Just under two-thirds of the 43 matched trial pairs used the same surrogate primary endpoints (28 of 43, 65.1%). There were six (14.0%) additional matched trials where the postapproval trial evaluated the same surrogate marker but the primary comparison was based on different intervention arms. Characteristics for the matched pivotal and postapproval trials, including intervention dosage, comparator, number of patients, and patient demographics, are included in Additional file 2.

Most approvals for which we identified matched pivotal and postapproval trials were for pharmacologic (small molecule) drugs for the treatment of chronic disease and were not granted orphan status, priority review, or approved through the accelerated approval pathway (Table 1). The most common therapeutic area was cardiovascular disease and diabetes mellitus (11 of 27). Compared with postapproval trials, pivotal trials had larger populations (*P* = 0.02), were more likely to be double or triple blinded (*P* = 0.008), and were less likely to have active only comparator arms (*P* = 0.04). Study duration and total number of treatment arms (including control) between the two groups of trials did not differ significantly (*P* = 0.57 and 0.14, respectively) (Table 2).

**Table 1:** Characteristics of novel drugs approved by the FDA based on pivotal trials using surrogate markers of disease between 2005 and 2012 with at least one matched controlled postapproval study

Characteristic	*N* (%)
Total indications	27 (100.0)
Agent type	
Pharmacologic	24 (88.9)
Biologic	3 (11.1)
Orphan status	
No	24 (88.9)
Yes	3 (11.1)
Approval pathway	
Regular	24 (88.9)
Accelerated	3 (11.1)
Priority review	
Standard	19 (70.4)
Priority	8 (29.6)
Therapeutic area	
Cardiovascular disease, diabetes mellitus	11 (40.7)
Infectious disease	6 (22.2)
Cancer	2 (7.4)
Other	8 (29.7)

**Table 2:** Characteristics of published, superiority, randomized, pivotal trials of novel drugs first approved by the FDA between 2005 and 2012 based on trials using surrogate markers of disease as primary endpoints with at least one postapproval match

	Pivotal trials	Postapproval trials	
Characteristics			*P* value^a^
Total studies	43 (100.0)	43 (100.0)	
Allocation			
Double or triple blind	39 (90.7)	31 (72.1)	0.008
Single blinded or open label	4 (9.3)	12 (27.9)	
Number of arms			
Median (interquartile range)	3 (2, 4)	2 (2, 4)	0.14^b^
Comparator options			
Active only	9 (20.9)	17 (39.5)	0.04
Placebo only or active and placebo	34 (79.1)	26 (61.5)	
Center status			
Multicenter	43 (100.0)	40 (93.0)	0.25
Single center	0	3 (7.0)	
Funder type			
Industry or mixed funding that includes industry	40 (93.0)	38 (88.4)	0.69
All others (non-profit, government, mixed non-industry, none, not specified)	3 (7.0)	5 (11.6)	
Sample size			
Total ITT or mITT, median (interquartile range)	672 (390, 822)	395 (154, 735)	0.02^b^
Study duration			
Duration in weeks, median (interquartile range)	24 (10, 26)	24 (12, 26)	0.57^b^

^a^Analyses based on McNemar’s exact test

^b^Analyses based on Wilcoxon signed-rank test

*ITT* intention to treat (when available, all subjects randomized), *mITT* modified intention to treat (all subjects randomized that received at least one treatment)

### Comparison of treatment effects

#### Pairs fulfilling at least two matching criteria (primary analyses)

A total of 86 premarket pivotal and postapproval trials were available for the paired analysis (43 matched pairs of trials evaluating the same drug and indication and using the same surrogate markers as endpoints). Since the results were similar using the different variance approximations, analyses were performed assuming that all matched pivotal and postapproval trials were independent. There were two matched pairs of trials that reported hazard ratios. However, based on the data provided, we assumed that these hazard ratios reasonably approximated odds ratios. Furthermore, there were four matched pairs fulfilling at least two matching criteria where the pivotal trial had a placebo comparator and the postapproval trial had an active comparator.

**Fig. 2:**
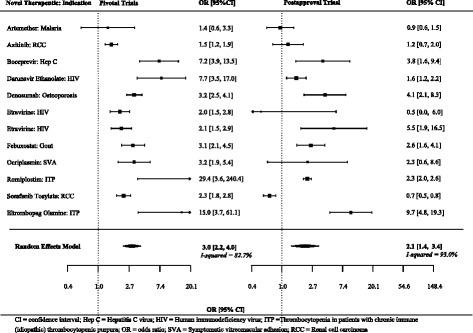
Individual and summary odds ratios from pivotal and postapproval trials fulfilling at least two matching criteria and reporting non-continuous endpoints

**Fig. 3:**
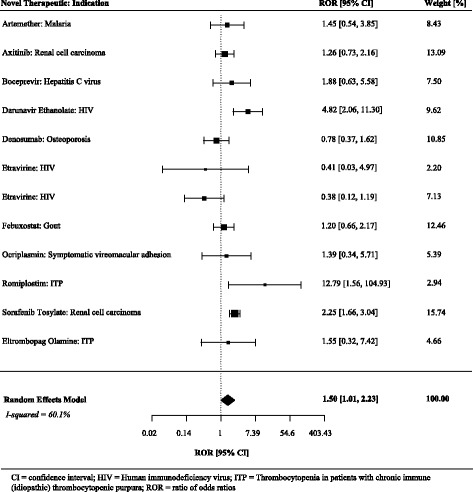
Ratios of odds ratios comparing pivotal and postapproval trials fulfilling at least two matching criteria and reporting non-continuous endpoints

The majority (*n* = 9, 75%) of the 12 ratios of odds ratios were greater than 1.0. On average, the treatment effects were 50% higher (more beneficial) in the pivotal trials than in the matched postapproval trials (summary ratio of odds ratios 1.50, 95% CI 1.01 to 2.23) (Figs. 2 and 3, Table 3).

**Table 3:** Comparison of treatment effects of pivotal trial using surrogate markers with matched postapproval trials using surrogate markers: primary and sensitivity analyses

	Summary difference between standardized mean differences or relative odds ratios (95% CI)
Method of analyses	
At least two matching criteria (*n* = 43)	
Reported non-continuous endpoints	1.50 (1.01 to 2.23)
Reported continuous endpoints	0.01 (−0.15 to 0.16)
All standardized as odds ratios (secondary)	1.12 (0.88 to 1.42)
At least three matching criteria (*n* = 39)	
Reported non-continuous endpoints	1.45 (0.99 to 2.14)^a^
Reported continuous endpoints	0.05 (−0.08 to 0.19)
All standardized as odds ratios (secondary)	1.17 (0.95 to 1.45)
All four matching criteria (*n* = 33)	
Reported non-continuous endpoints	1.21 (0.89 to 1.64)
Reported continuous endpoints	0.06 (−0.10 to 0.21)
All standardized as odds ratios (secondary)	1.13 (0.90 to 1.42)

A positive difference between standardized mean differences or ratio of odds ratios >1.0 indicates a larger benefit of treatment compared to the comparator in pivotal trials compared to postapproval trials. Pooled using DerSimonian and Laird random-effects meta-analyses

*CI* confidence interval

^a^95% CI of 1.00 to 2.10 with a between-study correlation of 0.5

**Fig. 4:**
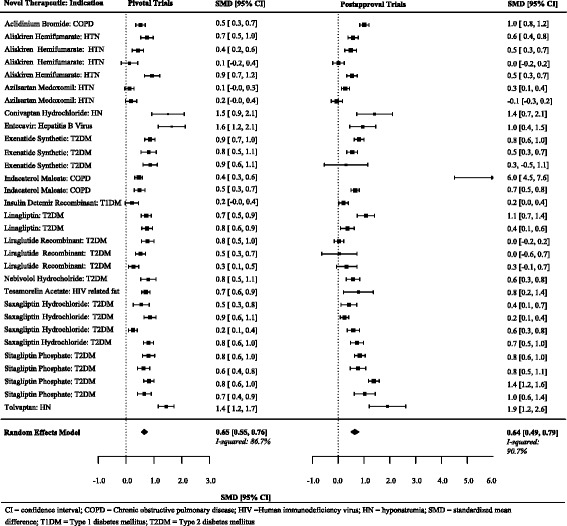
Individual and summary standardized mean differences from pivotal and postapproval trials fulfilling at least two matching criteria and reporting continuous endpoints

**Fig. 5:**
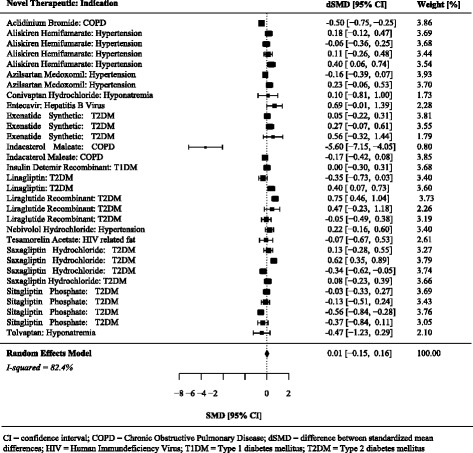
Differences between standardized mean differences comparing pivotal and postapproval trials fulfilling at least two matching criteria and reporting continuous endpoints

**Fig. 6:**
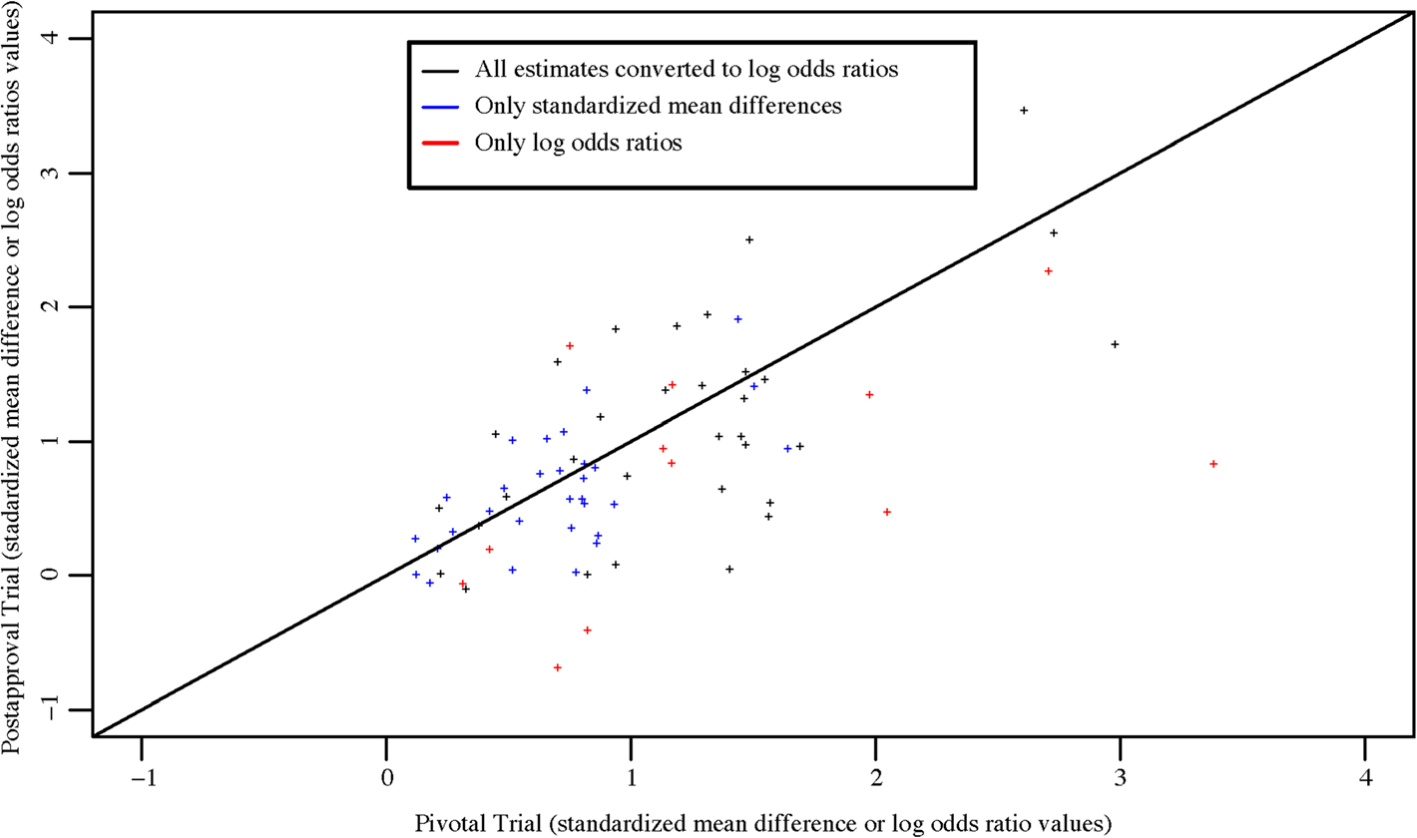
Comparison of surrogate-based effect estimates from pivotal trials and post approval trials. One outlier not show on graph

Just over half (*n* = 17, 54.8%) of 31 of the differences between the standardized mean differences were greater than 0.0. On average, there was no difference between standardized mean differences between the matched pivotal and postapproval trials (summary difference between standardized mean differences 0.01, 95% CI -0.15 to 0.16) (Figs. 4 and 5, Table 3), although Fig. 6 suggests there is a slight attenuation of continuous endpoints based on surrogate makers in postapproval trials.

#### Pairs fulfilling at least three or all four matching criteria

When the analyses were repeated among the 39 pairs of matched trials fulfilling at least three of the hierarchical matching criteria, the ratio of odds ratios focusing on trials with non-continuous surrogate markers of disease as trial endpoints appeared to be somewhat attenuated toward the null (summary ratio of odds ratios 1.45, 95% CI 0.99 to 2.14). The pooled standardized mean differences from pivotal trials were 0.05 higher (more beneficial) compared to the matched postapproval trials reporting continuous endpoints based on surrogate markers (summary difference between standardized mean differences 0.05, 95% CI -0.08 to 0.19). The findings were similar when only the 33 matched pairs fulfilling all four of the matching criteria were considered (Table 3).

#### Secondary analyses

After converting all of the standardized mean differences to odds ratios, we estimated the ratio of odds ratios for all 43 matched pairs. On average, the treatment effects were 12% higher (more beneficial) in the pivotal trials reporting endpoints based on surrogate markers than in the postapproval trials reporting endpoints based on surrogate makers (summary ratio of odds ratios 1.12, 95% CI 0.88 to 1.42). Results were similar when analyses were repeated for matched pairs that fulfilled at least three or all four of matching criteria (Table 3).

#### Exploratory analyses: postapproval trials using patient-relevant outcomes

We located three novel drugs for three indications based on pivotal trials using surrogate markers as primary endpoints that could be matched to postapproval trials evaluating the same drug and indication but using patient-relevant outcomes as endpoints, for a total of three matches, which is an insufficient number for a meta-analysis. However, the individual odds ratios calculated from the pivotal trials based on surrogate markers were between 160% and 1180% larger than the odds ratios calculated from the postapproval trials based on patient-relevant outcomes. One full comparison is described in Box 1 for illustrative purposes and the remaining comparisons can be found in Additional file 3.

## Discussion

Our study found that the treatment effects based on non-continuous surrogate markers in pivotal trials are larger than, and may overestimate, the treatment effects based on the exact same non-continuous surrogate markers when used in postapproval trials. Although we did not observe any statistically significant differences when we compared treatment effects observed based on continuous surrogate markers, our findings highlight the potential problem in the estimation of the magnitude of treatment effects of drugs that are approved based on surrogate markers of disease. Furthermore, we found that many postapproval drug trials are not directly comparable to previously published pivotal trials, particularly with respect to endpoint selection. In the exploratory analyses, we found that the three postapproval trials with patient-relevant outcomes had smaller treatment effects than the pivotal trials using surrogate markers of disease as trial endpoints.

Several reasons may explain why pivotal trials could have larger treatment effects based on surrogate markers compared with postapproval trials. First, pivotal trials may have more stringent inclusion criteria, which could lead to larger treatment effects, particularly if sicker or less homogeneous patient populations are included in trials. Evidence suggests that pivotal trials underrepresent certain groups, including elderly patients and those from racial and ethnic minorities [26]. Furthermore, premarket pivotal randomized controlled trials may be more likely to exclude patients taking common medications and those with certain comorbidities [27]. Postapproval trials, with potentially more relaxed patient eligibility standards, may have attenuated treatment effects compared to pivotal trials if they evaluate drugs in expanded populations or in combination with other interventions. Second, our findings may coincide with the observation that most large treatment effects become smaller when additional studies are performed and evidence is accumulated [15]. The data observed in one pivotal trial may be at the far extreme of a distribution of all possible treatment effects. Third, study selection bias could explain why pivotal trials have large treatment effect estimates. It is possible that controlled trials with the largest treatment effects, selected from among all studies submitted to the FDA, are more likely to be designated as essential (pivotal) during the drug approval process. It is also possible that our finding of a statistically significant difference between treatment effects from pivotal and postapproval trials is spurious. In particular, we approximated odds ratios for all non-continuous surrogate markers, which are known to be unstable when sample sizes are small.

Even though we found that pivotal trials reported larger treatment effects when compared with postapproval trials of non-continuous surrogate markers of disease as trial endpoints, we did not observe any statistically significant differences when we focused on continuous surrogate markers. Overall, we are uncertain why there were differences between continuous and non-continuous surrogate markers. While our findings could suggest that the evidence used to support the approval of novel therapeutics by the FDA is reliable and potentially consistent in postapproval trials evaluating the same drugs in diverse populations, it is also possible that the treatment effects reported in postapproval trials are exaggerated. It can be argued that pivotal trials, which evaluate the efficacy of novel therapeutics for the first time, face lower publication standards compared to postapproval trials. The authors of postapproval trials evaluating primary endpoints based on the same surrogate makers as previously published pivotal trials may submit only manuscripts with large statistically significant treatment effects to improve the chances of getting published. As a result, the differences between treatment effects observed in our analyses could be underestimated. In our sample, postapproval trials were also smaller and less likely to be double or triple blinded, which may indicate lower methodological quality compared to pivotal trials. Previous evidence suggests that lack or unclear double-blinding is associated with an average of 13% exaggeration of treatment effects (ratio of odds ratios 0.87; 95% CI 0.79 to 0.96) [13], and that small trials typically have larger treatment effects [13, 15, 28–30]. Exaggerated postapproval treatment effects due to lower methodological study quality could explain why we did not observe statistically significant differences between the treatment effects in many of our analyses.

Finally, postapproval trials using patient-relevant outcomes were unlikely to evaluate the exact same intervention in comparison to the exact same active or placebo control as the pivotal trials with endpoints based on surrogate markers. To compare continuous and non-continuous treatment effects for our exploratory analyses, we used a common approach that allowed for conversion from standardized mean differences to odds ratios [24, 25]. In meta-analyses and meta-epidemiological evaluations, not all studies use the same kind of data. Our transformations were also informed by a previously published meta-epidemiological study comparing continuous and non-continuous outcomes [6]. Although this method leads to some loss of power [25], we found that the pivotal trials with endpoints based on surrogate markers reported larger treatment effects than the three postapproval trials using patient-relevant outcomes. In a prior study of 324 consecutive cardiovascular trials published in major general medical journals between 2000 and 2005, trials reporting surrogate primary outcomes were more likely to report a positive treatment effect than trials reporting patient-relevant primary outcomes [31]. Evidence also suggests that trials with large effects are more likely than other trials to use laboratory-defined efficacy, such as endpoints based on surrogate markers [15]. Similarly, a recent meta-epidemiological study of randomized clinical trials published in 2005 and 2006 in six high impact medical journals found that trials with surrogate primary outcomes report larger treatment effects than trials reporting patient-relevant primary outcomes [6].

### Implications of the study

Over the last few years, there have been new proposals for increased reliance on smaller and shorter trials with wider use of surrogate markers in the United States [32]. The pressure to use surrogate markers has already resulted in policy proposals. For instance, the *21st Century Cures Act*, which was introduced in the U.S. House of Representatives in May 2015, encouraged the FDA to rely more on surrogate markers instead of patient-relevant outcomes [32]. In 2006, the Institute of Medicine Report on the future of drug safety recommended that the FDA monitor and evaluate the benefits and risk of drug therapies throughout their entire market life [33]. With this lifecycle evaluation approach, drugs can be approved based on limited evidence, with the understanding that they will continue to be evaluated after approval. We found that when postapproval trials do evaluate surrogate markers, the treatment effects are smaller in comparison to the treatment effects observed among pivotal trials using the same endpoints based on surrogate markers of disease, specifically for non-continuous surrogate markers. This may mislead payers, policymakers, patients, and physicians who need to determine the benefit of a medication in clinical practice, particularly in comparison to other available drugs and therapeutic approaches. If pivotal trials using surrogate markers of disease as trial endpoints have larger treatment effects that result from their stringent eligibility criteria, efforts are necessary to ensure that pivotal trials evaluate novel therapeutics in more diverse patient populations. Before promoting the broader use of surrogate markers, an FDA advisory committee may be necessary to examine why the same surrogate markers for the same drugs do not always replicate exactly in postapproval studies. Requirements for postapproval trials should also be enhanced to strengthen the lifecyle evaluation. In particular, investigators should conduct postapproval studies that have adequate sample sizes, study durations, and design characteristics, including endpoints, to allow comparison to pivotal trials. The FDA may need to enhance their enforcement of formal empirical verification of surrogate markers, which can help to establish the performance of a surrogate marker and decide whether it should continue to be used as a substitute and predictor of treatment benefit [8]. Lastly, the FDA should continue to ensure that sponsors complete postmarketing requirements (PMRs). Evidence suggests that although sponsors are completing most PMRs to schedule, certain PMRs are delayed and the FDA continues to have problems with its ability to track PMRs [34].

### Limitations

Our study has certain limitations. First, we limited our analysis to drugs evaluated by the FDA. Our results may not be generalizable to other regulatory authorities, including the European Medicine Agency, Health Canada, and Japan’s Pharmaceutical and Medical Devices Agency.

Second, we restricted our analysis to studies that had been published in the biomedical literature and indexed in Medline. While this search may have excluded some potential postapproval trials, it is unlikely that we missed many studies not indexed in Medline that are used to inform clinical practice [17].

Third, we attempted to maximize the comparability between pivotal and postapproval trials by using a hierarchical matching process. To ensure an adequate number of matches, we required only that pivotal and postapproval trials evaluated the same drug for the same indication with the same endpoint based on a surrogate marker. Even though the majority of our sample fulfilled all four of the matching criteria, most matched trials will have different eligibility criteria and/or methodological characteristics, which can influence the treatment effects observed. Furthermore, we did not consider the potential co-treatment effects when additional drugs were studied that may have affected treatment effect sizes. However, if additional matching criteria had been used, the number of comparable pivotal and postapproval matches would have been reduced. Furthermore, when the analyses were repeated among the 33 matched pairs fulfilling all four of the matching criteria, the ratio of odds ratios focusing on trials with non-continuous surrogate markers of disease as trial endpoints appeared to be somewhat attenuated toward the null. This could suggest that the different comparator groups (e.g., placebo or active control) may influence the magnitude of the primary findings.

Fourth, with only 12 matched pivotal and postapproval trials with non-continuous surrogate markers of disease as trial endpoints in our sample, our findings are subject to uncertainty. Even though odds ratios are one of the most commonly reported measures of association or effect, relative risk calculations are unstable when sample sizes are small.

Fifth, trials are generally powered to detect statistically significant differences in their primary endpoint. Although the endpoints based on surrogate markers in the pivotal trials were all primary outcomes, to maximize the number of matches, we considered endpoints based on surrogate markers that were not the primary endpoint reported in the postapproval trials. As a result, postapproval trials may not have been powered to detect treatment effects based on the secondary surrogate markers of interest.

Lastly, to locate an adequate number of matches, we considered certain postapproval trials where it was unclear whether they were performing superiority or non-inferiority analyses.

## Conclusions

The treatment effects from pivotal trials supporting FDA approval of novel therapeutics based on surrogate markers of disease are often larger than the treatment effects observed among postapproval trials using surrogate markers as trial endpoints, specifically for non-continuous surrogate markers. New proposals for increased reliance on smaller and shorter trials with surrogate markers carry the risk of demonstrating larger, and potentially exaggerated, treatment effects, which may ultimately lack reproducibility or generalizability. Policymakers, doctors, and patients should interpret treatment effects based on surrogate markers of disease as primary endpoints with caution, and in the absence of clinical outcomes, regulators and payers should focus on those surrogate markers that have been validated.

## Box 1: Illustrative example of a postapproval trial using a patient-relevant outcome

***Pivotal trials***

In 2007, the FDA approved Tekturna (aliskiren) 150 mg and 300 mg tablets for the treatment of hypertension. The approval was based on five randomized double-blind placebo-controlled pivotal trials evaluating the reduction in mean sitting diastolic blood pressure (msDBP), a surrogate marker of disease, as the primary efficacy outcome. For all five trials, we focused on the comparison of aliskiren 300 mg with the placebo. We extracted absolute changes in msDBP and calculated standardized mean differences between treatment and placebo for all five studies. After standardizing the standardized mean differences so that a positive value (greater than 0.0) indicates a beneficial effect of intervention, we transformed the standardized mean differences to log odds ratios and combined them using the DerSimonian and Laird procedure for random effects. We found a summary odds ratio of 3.11 (95% CI 2.21 to 4.36), indicating the superiority of aliskiren in terms of reducing msDBP.

***Postapproval trials***

There was one randomized postapproval study that compared aliskiren (300 mg) with a placebo for the treatment of hypertension that evaluated a patient-relevant outcome—a composite endpoint of cardiovascular death, non-fatal myocardial infarction, non-fatal stroke, and clinically significant heart failure. Using the data available in the publication, we calculated an odds ratio of 0.82 (95% CI 0.37 to 1.81). This odds ratio was then transformed so that a value above 1.0 indicates a beneficial effect of intervention (odds ratio 1.22; 95% CI 0.55 to 2.70).

***Comparison of odds ratios***

After calculating the ratio of odds ratios, we found that the treatment effects were 160% higher in the five pivotal trials using surrogate markers than in the one postapproval trial using a patient-relevant outcome (ratio of odds ratios 2.6; 95% CI 1.05 to 6.23).

## Additional files

Additional file 1: Study protocol. (PDF 145 kb)

Additional file 2: Data. Matched pivotal and postapproval trials. (XLSX 36 kb)

Additional file 3: **Table S1.** Pivotal trials with at least one postapproval trial with a patient-relevant outcome. (XLSX 31 kb)

## Abbreviations

CI: Confidence interval
COPD: Chronic obstructive pulmonary disease
dSMD: Difference between standardized mean differences
FDA: Food and Drug Administration
Hep C: Hepatitis C virus
HIV: Human immunodeficiency virus
HN: Hyponatremia
ITP: Idiopathic thrombocytopenic purpura
msDBP: mean sitting diastolic blood pressure
OR: Odds ratio
PMR: Postmarketing requirement
RCC: Renal cell carcinoma
ROR: Ratio of odds ratios
SMD: Standardized mean difference
SVA: Symptomatic vitreomacular adhesion
T1DM: Type 1 diabetes mellitus
T2DM: Type 2 diabetes mellitus
