# Supplementary material for: Comparison of treatment effect sizes from pivotal and postapproval trials of novel therapeutics approved by the FDA based on surrogate markers of disease: a meta-epidemiological study
Source: BMC Med. 2018 Mar 21;16:45. doi: 10.1186/s12916-018-1023-9 (PMC5863466; doi:10.1186/s12916-018-1023-9)
Supplement: Supplementary file 1 — Study protocol. (PDF 145 kb) [file 12916_2018_1023_MOESM1_ESM.pdf]

Study Protocol

Comparison of treatment effect sizes from pivotal and postapproval trials of novel therapeutics approved by the FDA on the basis of surrogate markers of disease

Joshua D Wallach, Oriana Ciani, Alison Pease, Gregg Gonsalves, Harlan M Krumholz, Rod S Taylor, Joseph S Ross

Correspondence to: Joshua D Wallach, MS, PhD, Collaboration for Research Integrity and Transparency (CRIT), Yale Law School; and Center for Outcomes Research and Evaluation, Yale-New Haven Health System, New Haven, CT, USA
Phone: 510-575-9130, Email: joshua.wallach@yale.edu

Specific aims:

Aim: To quantify and compare the treatment effects from pivotal trials of novel therapeutics approved by the FDA on the basis of surrogate end points to the treatment effect sizes from postapproval trials, matching for the same indication, outcome, treatment dosage, and comparator.

> **Sub aim 1**: To determine whether any differences that may exist between treatment effect sizes from pivotal trials and the treatment effect sizes from postapproval trials, matching for the same indication, outcome, treatment/comparator, and treatment dosage, may be explained by study sample size, center status (multicenter, single centers), and follow-up time.
>
> **Protocol modification 1:**
>
> **These analyses were not pursued as of yet. After completing the primary aim, we had enough data to interpret and discuss.**

METHODS

Identification of pivotal trials

To identify pivotal trials using surrogate markers as their primary outcome for novel therapeutic agents, we used previously collected data.[1] The database contains information about novel therapeutics first approved by the FDA between January 1, 2005, and December 31, 2012. The Drugs@FDA database was used to categorize each novel therapeutic agent by year of approval and as a pharmacologic entity (small molecule) or biologic. FDA approval letters, which are hyperlinked in the Drugs@FDA database, were then used to determine the indications for which all novel therapeutic agents were initially approved for use, whether agents were orphan drugs, and whether agents were approved through the accelerated approval pathway. The World Health Organization's Anatomic Therapeutic Classification system was used to categorize each indication into therapeutic areas (cancer, cardiovascular disease and diabetes mellitus, infectious disease, and other).[2] Primary trial endpoints were classified as clinical outcomes, clinical scales, or surrogate outcomes based on an established framework and an Institute of Medicine report.[3 4] Clinical outcomes (ie, mortality) represent patient survival or function, clinical scales (ie, Crohn's Disease Activity Index) represent the quantification of subjective patient-reported symptoms, and surrogate markers (ie, changes in blood pressure) represent biomarkers expected to predict clinical benefit. Study descriptions, additional definitions, and inclusions and exclusion criteria appear in the original publication.[1]

We did not consider additional novel therapeutics approved after December 31, 2012 because insufficient time has passed since approval to allow for completion and publication of post-approval trials. Three to

four years may not be long enough for a new randomized controlled trial to publish a corroboration attempt for the same indication with the same therapeutic and surrogate marker of disease.[5]

To identify publications of pivotal efficacy trials for novel therapeutic agents approved between 2005 and 2011, we also used previously collected data.[6] Briefly, the biomedical literature during the period from April through October 2012 was searched. In particular, the Scopus database (Elsevier Inc) was searched using the terms "[generic drug name]" AND "clinical trial" and when necessary, the manufacturer-designated trial identification numbers of 6 or more characters were entered into the advanced search feature of ClinicalTrials.gov. Four criteria were used to identify matching publications: study design, indication, intervention, and intention-to-treat enrollment. One author (JDW) performed additional searches to locate the novel therapeutic agents approved in 2012. Detailed descriptions appear in the original research letter.[6]

Identification of postapproval trials

The International Nonproprietary Name of each drug approved by the FDA between January 1, 2005 and December 31, 2012 were searched in PubMed to locate all English-language publications describing postapproval human subject studies of the novel therapeutic agents that used an active or placebo control as a comparator arm and examined efficacy for the same therapeutic indication for which the drug was original approved by the FDA, as described in previous work.[7] The primary trial endpoints of eligible postapproval studies were then classified as clinical outcomes, clinical scales, or surrogate markers based on an established framework and a recent Institute of Medicine report.[3 4] Medline was utilized because it is the largest database of biomedical journal articles that can be searched freely using the PubMed system. Furthermore, the vast majority of doctors and policy makers rely on the PubMed system to learn about clinical trial findings. Study descriptions, additional definitions, and inclusions and exclusion criteria appear in the original manuscript.[7]

Study selection

One author (JDW) undertook the inclusion, exclusion, and matching of pivotal and postapproval trials. We excluded pivotal and postapproval trials that (1) were not published; (2) were not interventional, randomized trials; (3) had equivalence or non-inferiority design; (4) only had one arm (i.e., no comparator groups); (5) had mixed primary outcomes (i.e., composite endpoints where both surrogate and final end points are included);[A] (6) were crossover trials; and (7) had no analyzable data. We further excluded postapproval studies that only had treatment arms where the novel therapeutic of interested was combined with other active interventions not considered in any of the corresponding pivotal trials. Although

individual pivotal trial results are available in the FDA medical reviews on the drugs@FDA database, our study focused on the pivotal trial data published in peer reviewed biomedical journals. This allowed for matched pairs of published pivotal and postapproval trials. Potential matches and uncertainties were discussed with an additional investigator (JSR).

Protocol addition:

[A] None of the trials that we evaluated had mixed primary outcomes, so this exclusion criteria was not mentioned in the final manuscript.

Protocol modification 2:

During the data extraction process, we discovered that there were few postapproval trials using patient relevant outcomes. We updated our protocol to reflect that most postapproval trials evaluated surrogate markers of disease as primary endpoints:

"When postapproval trials used patient relevant outcomes for the primary or secondary trial endpoint, a successful match of a pivotal and postapproval trial required that they each evaluated the same drug for the same indication. For potential matches, we further identified whether the matched trials evaluated the same intervention dosage and the same comparator (ie, placebo, usual care, or active comparator)."

Due to these changes, all analyses involving postapproval trials with patient relevant outcomes were considered exploratory.

Matching pivotal trials with post-approval trials

To create a sample of comparable published pivotal trials and postapproval trials, one investigator (JDW) used a hierarchical matching process to match the individual pivotal trial for each drug-indication with one postapproval randomized controlled trial based on the following four criteria: use of the same (1) novel therapeutic for the same indication; (2) surrogate marker that was the primary outcome in the pivotal trial(s) used to form the exclusive basis of approval by the FDA; (3) intervention dosage; and (4) comparator (ie, placebo or active comparator). At a minimum, matched pivotal and postapproval trials were required to evaluate the same drug for the same indication and the same surrogate marker outcome (criteria (1) and (2)). For criteria (2), we allowed some flexibility in terms of timing (ie, a pivotal trial with sustained virologic response (SVR) at week 24 could be matched with a postapproval trial with SVR at week 12) and how the outcomes were measured (ie, the time of day measurement was taken). For

dosage, we looked for treatment arms in the pivotal and postapproval trials with the exact same therapeutic dosage (ie, a pivotal trial evaluating 750 mg of telaprevir two times a day could be matched with a postapproval trial evaluating 1500 mg one time a day), but did not require the timing of the treatment (ie, multiple injections provided 7-9 hours apart) or the length of treatment (ie, 12 weeks vs. 24 weeks) to be exactly the same. We allowed some flexibility in terms of background therapies in the pivotal and postapproval matches (ie, a pivotal trial evaluating liraglutide in combination with metformin and thiazolidinedione could be matched with a postapproval trial evaluating liraglutide in combination with metformin only). When possible, we attempted to match pivotal and postapproval trials that used the same comparator arm. When pivotal trials only had a placebo comparator and postapproval trials only had active comparators, we select the comparator arm in the postapproval trial with the lowest dosage. When pivotal trials had multiple postapproval trial matches, we selected the trial with the longest follow-up time and the largest number of intention to treat patients in the intervention and comparator arms.

Data extraction

For each novel therapeutic, we recorded the indication for which all novel therapeutics agents were initially approved for use and the therapeutic area (based on the World Health Organization's Anatomic Therapeutic Classification system). We recorded whether the novel drugs were pharmacologic entities (small molecule) or biologics; were classified as having orphan status; or were approved through the accelerated approval pathway. For pivotal trials and postapproval studies, we recorded: total sample size (intention to treat)[B]; trial duration (in weeks); center status (multicenter, single centers); funding (for profit, not for profit, mixed, or none); subject allocation (i.e., double-blind, single-blind, or open label); and comparator type (i.e., placebo only, active only, or both).[C]

Protocol additions:

[B] **Total sample size (intention to treat (ITT), all subjects initially randomized or modified intention to treat (mITT), all subjects randomized that received at least one treatment).**

[C] **We also extracted certain demographic characteristics (% female, % non-Caucasian, and mean or median age of study subjects).**

For all of the published pivotal and matched postapproval trials, we extracted the number of patients and events in the selected treatment and control arms, the absolute or relative effect sizes, confidence intervals (CIs), standard deviations, standard errors, or any other available data to calculate the endpoints based on surrogate markers or clinical outcomes. When necessary, an online digitizer (Web-PlotDigitizer) was used

to extract approximate values from figures. Lastly, we recorded whether the matched trial pairs fulfilled 2, 3, or 4 of the matching criteria.

Data analyses

We used descriptive statistics to characterize the eligible novel drugs approved by the FDA and to summarize the design features of the pivotal trials and matched postapproval trials. We used Wilcoxon's signed rank and McNemar's exact tests to examine differences between matched pairs. All descriptive analyses were performed by one investigator (JDW) using SAS (version 9.4, SAS Institute; Cary, NC).[D] All statistical tests were two tailed and used a type 1 error rate of 0.05.

Protocol addition:

[D] Meta-analyses were performed using the metafor package in R (version 3.2.3; The R Project for Statistical Computing)

We compared the treatment effects between pivotal and postapproval trials using several analytical approaches. For our primary analysis of trials fulfilling the first two matching criteria, we first calculated standardized mean differences (Cohen's *d*) for trials with continuous outcomes and odds ratios for trials reporting counts, proportions, or relative effect estimates (ie, we calculated a standardized mean difference when a study reported a mean difference and an odds ratio when a study reported a hazard ratio). The direction of effect was standardized so that an odds ratio above 1.0 or standardized mean difference above 0.0 indicated a beneficial effect of intervention compared to active or placebo arms. We first combined effect estimates separately across pivotal and postapproval trials using the DerSimonian and Laird procedure for random effects. For each matched pair with a continuous endpoint based on a surrogate marker, we then estimated paired differences between standardized mean differences and associated standard errors. A difference between standardized mean differences greater than 0.0 implied greater (more beneficial) treatment effects in the pivotal trials using a surrogate marker than in the postapproval trials. For matched pairs with trials reporting counts, proportions, or a relative effect estimate, we first converted odds ratios to natural log odds ratios and then calculated the ratio of odds ratios, using the method of Bucher et al.[8] Differences between standardized mean differences or ratios of odds ratios were combined using the DerSimonian and Laird procedure for random effects. All analyses were also repeated for pivotal and postapproval matches that fulfilled at least three or all four of the matching criteria. Meta-epidemiological decisions and uncertainties were discussed with an additional investigator (OS).

Protocol modification 3:

Based on the protocol modification 2, we also updated the data analyses section of our protocol:

Primary analyses

When pivotal trials were matched only to postapproval trial using surrogate markers of disease for one of the trial endpoints, we first separately combined the standardized mean differences and odds ratios across pivotal and postapproval trials using the DerSimonian and Laird procedure for random effects. We performed our analyses under the random-effects meta-analysis model assumptions. In particular, that the true treatment effect might be different between individual trials (eg, treatment effects could be higher among trials with older or less healthy patients).[9] For each matched pair with a continuous endpoint based on a surrogate marker, we then estimated paired differences between standardized mean differences. For each matched pair with non-continuous surrogate markers of disease as trial endpoints, we converted odds ratios to natural log odds ratios and then calculated the ratio of odds ratios, using the method of Bucher et al.[8] A positive difference between standardized mean differences (greater than 0.0) and a ratio of odds ratios greater than 1.0 implied greater (more beneficial) treatment effects in the pivotal trials using a surrogate marker than in the postapproval trials using a surrogate marker. Considering that individual pivotal and postapproval trials were matched based on 2 to 4 criteria, we calculated the variance of each individual ratio of odds ratios using two methods: (1) assuming that the pivotal and postapproval trials in the matched pairs were independent and (2) assuming between study correlations of 0.5. If different results were observed using the two methods, we planned to repeat the calculations using correlation coefficients of 0.2, 0.3, and 0.4. If similar results were observed using the different variance approximations, we reported the more conservative estimates (assuming that all matched pivotal and postapproval trials were independent). Differences between standardized mean differences and ratios of odds ratios were separately combined using the DerSimonian and Laird procedure for random effects. All analyses were repeated for pivotal and postapproval trial matches that fulfilled at least 3 or all 4 of the matching criteria.

Secondary analyses

Standardized mean differences and associated variances for all pivotal and postapproval trials reporting continuous endpoints were transformed to natural log odds ratios.[10] The ratio of odds ratios from all matched pairs fulling 2, 3, or all 4 matching criteria were then combined using the DerSimonian and Laird procedure for random effects.

Exploratory analyses

When pivotal trials were matched to postapproval trial using patient relevant outcomes for one of the trial endpoints, the standardized mean differences from the pivotal trials were transformed to natural log odds ratios.[10] We then calculated the ratio of odds ratios using the method of Bucher et al.[8] The paired ratios of odds ratios were then combined using the DerSimonian and Laird procedure for random effects. Ratios of odds ratios greater than 1.0 implied greater (more beneficial) treatment effects in the pivotal trials than in the postapproval trials. All variances were calculated as described above.

From original protocol:

Sensitivity Analyses **(same as secondary analyses in the modified section above)**

Standardized mean differences and associated standard errors for all trials reporting continuous outcomes were transformed to natural log odds ratios. The ratio of odds ratios from all matched pairs fulling 2, 3, or all 4 matching criteria were then combined using the DerSimonian and Laird procedure for random effects.[10]

Patient involvement

No patients were involved in setting the research question or the outcome measures, nor were patients involved in any other aspect of study design or implementation.

REFERENCES

1. Downing NS, Aminawung JA, Shah ND, et al. Clinical trial evidence supporting FDA approval of novel therapeutic agents, 2005-2012. *JAMA* 2014;311(4):368-77. doi: 10.1001/jama.2013.282034
2. World Health Organization (WHO) Collaborating Center for Drug Statistics Methodology. ATC/DDD index 2017. https://www.whocc.no/atc_ddd_index/ .
3. Institute of Medicine. Ethical and Scientific Issues in Studying the Safety of Approved Drugs. 2012.
4. Clement FM, Harris A, Li JJ, et al. Using effectiveness and cost-effectiveness to make drug coverage decisions: a comparison of Britain, Australia, and Canada. *JAMA* 2009;302(13):1437-43. doi: 10.1001/jama.2009.1409
5. Wallach JD, Sullivan PG, Trepanowski JF, et al. Evaluation of Evidence of Statistical Support and Corroboration of Subgroup Claims in Randomized Clinical Trials. *JAMA Intern Med* 2017 doi: 10.1001/jamainternmed.2016.9125 [published Online First: 2017/02/13]

6. Smithy JW, Downing NS, Ross JS. Publication of pivotal efficacy trials for novel therapeutic agents approved between 2005 and 2011: a cross-sectional study. *JAMA Intern Med* 2014;174(9):1518-20. doi: 10.1001/jamainternmed.2014.3438
7. Pease AM, Krumholz HM, Downing NS, et al. Postapproval studies of drugs initially approved by the FDA on the basis of limited evidence: systematic review. *BMJ* 2017;357:j1680. [published Online First: 2017/05/03]
8. Bucher HC, Guyatt GH, Griffith LE, et al. The results of direct and indirect treatment comparisons in meta-analysis of randomized controlled trials. *J Clin Epidemiol* 1997;50(6):683-91.
9. Borenstein M, Hedges L, Higgins P, et al. Introduction to Meta-Analysis: John Wiley & Sons, Ltd 2009.
10. Chinn S. A simple method for converting an odds ratio to effect size for use in meta-analysis. *Stat Med* 2000;19(22):3127-31.
